# Single- and Multi-Network Hydrogels for Soft Electronics—A Review

**DOI:** 10.3390/gels11070480

**Published:** 2025-06-21

**Authors:** Md Murshed Bhuyan, Nahid Hasan, Jae-Ho Jeong

**Affiliations:** 1 Department of Mechanical, Smart, and Industrial Engineering (Mechanical Engineering Major), Gachon University, 1342, Seongnam-daero, Sujeong-gu, Seongnam-si 13120, Republic of Korea; 2 School of Mechanical Engineering, College of Engineering, Chung-Ang University, 84 Heukseok-ro, Dongjak-gu, Seoul 06974, Republic of Korea

**Keywords:** soft electronics, flexibility, hydrogel, conductivity, biocompatible

## Abstract

Soft or flexible electronics is a rapidly growing and pioneering research field, as it makes devices comfortable to use, especially in biomedical engineering. Both single- and multi-network hydrogels have diverse applications where the most significant one is in the building of soft electronics, including soft circuits, displays, sensors, batteries, and supercapacitors, electronic storage, electric skin, health monitoring devices, soft robots, and automotive. Three-dimensional printing of conductive gels/hydrogels facilitates the construction of soft electronics. This review illustrates the design, mechanism, and application of hydrogel in soft electronics. The current progress, scope of improvement, and future prospects of hydrogel-based soft electronics are also discussed. This review will provide a clear concept of the topic to researchers.

## 1. Introduction

Electronics having suitable softness and flexibility required for comfortable utility are referred to as soft electronics. Flexibility, or stretchability, is needed for reliable and long-term performance, especially in health monitoring [1]. Soft electronics are widely used in sensors, electrodes, energy harvesters, health monitoring, and robots [2,3]. Solid metals and semimetals, which have excellent optoelectric properties, are used to make conventional electronics, but they lack mechanical strength and flexibility. Owing to their superior mechanical compliance and electronic and optical properties, the materials used to prepare soft electronics are carbon nanotubes, silver nanowires, copper nanowires, gold nanowires, ZnO nanowires, hydrogels, liquid metals, polymer nanowires, graphene, and hydride nanowires [4,5,6]. A soft semi-solid material that consists of two or more monomers or polymers, or a monomer–polymer combination through physical or chemical bonding, that can hold solvent without dissolving in it, is referred to as a gel. If the gel can retain water 100 times their dried weight, then it is called a hydrogel [7]. A hydrogel can form a three-dimensional network with a void space where the foreign particles (solvent, metal, dyes, etc.) can be adsorbed through physical or chemical bonding [8,9]. A single-network hydrogel is brittle and has weak mechanical properties, and is prepared from the cross-linking of a single polymer like pectin, cellulose, polyacrylamide (PAAm), and polyethylene oxide, etc., with a cross-linking agent. In a multi-network hydrogel, two or more distinct polymers entangle through cross-linking/grafting, possessing strong mechanical strength and tunable properties. Single-network hydrogels (SN gels) are composed of a single type of hydrophilic polymer network, which is typically homogeneous, leading to a uniform distribution of mechanical properties. However, they are generally soft, weak, and brittle, failing under low tensile stress and strain. In contrast, double- or multi-network hydrogels (DN/MN gels) consist of two or more interpenetrating polymer networks where the first network is a rigid, highly cross-linked polyelectrolyte, while the second/third is a more ductile, poorly cross-linked neutral polymer. This heterogeneity contributes to their superior mechanical properties [10]. Both single- and multi-network hydrogels can be applied in soft electronics [11,12]. Hydrogels possess some extraordinary properties, such as stimuli responsiveness, flexibility, structure mimicking, and functions of living bodies, such as biodegradability and biocompatibility, self-healing, conductivity, and responsiveness to biological signals, which facilitate their use as vital parts of soft electronics as well as bioelectronics. On the basis of properties, a specific gel/hydrogel is selected to use in electronic devices [13,14]. The comparison between traditional and hydrogel-based soft electronics is presented in Table 1. Daihui Zhang et al. developed a cellulose-based hydrogel mimicking skin for use in a stable strain sensor to monitor human motions [15]. Soft electronics that need conductive hydrogels are fabricated using conductive polymers like poly (3,4-ethylenedioxythiophene: polystyrene sulfonate) (PEDOT: PSS), polyaniline (PANI), polypyrrole (PPy), polythiophene (PTh), and polycarbazole (PC). Non-conductive hydrogels with stretchability, softness, and conformability can also be used in soft electronics; for example, polyacrylamide, poly(vinyl alcohol), tannic acid, and chitosan-based hydrogels are used as insulators and interfaces [16,17]. Conductive hydrogels are categorized into three types: (i) ionic conductive hydrogels, (ii) electroconductive hydrogels, and (iii) metal-based conductive hydrogels. Since the initial “Sensitive Skin Workshop” in 1999, researchers have made substantial progress in the manufacture of flexible electronics. Since then, several research projects have been conducted to improve the performance of hydrogel in soft electronics by incorporating carbon nanotubes, graphene, ionic liquids, and inorganic fillers [18]. A revolution came when nano single- and multi-network hydrogels were implemented in the building of soft electronics [19]. For example, Jun Ho Lim et al. photo-polymerized choline chloride-acrylic acid (CA) deep eutectic solvents (DESs) with poly(3,4-ethylenedioxythiophene): poly (styrene sulfonate) (PEDOT: PSS) to prepare choline chloride-acrylic acid-PEDOT: PSS (CAP) conductor nanogel networks [20]. Owing to H-bonding in the structure, it is stretchable and self-healable and can be used in wearable soft electronics [20]. Despite hydrogels playing significant roles in the modern electronics industry, they currently have certain limitations, including low conductivity, limited mechanical strength and durability, degradation after certain use cycles, and some toxicity. Under high stress, biocompatible hydrogel electronics may undergo structural breakdown, and, at high or low temperatures, the water inside hydrogel networks may evaporate or freeze, which would reduce the device’s mechanical stretchability and performance. Further research can be conducted to improve the above-mentioned drawbacks. In this review, detailed explanations of the functional mechanisms of hydrogels in various soft electronics are presented with figures.

## 2. Hydrogel-Based Sensors

The word “sensor” comes from the Latin word “sensus”, which literally translates to “sense” or “to sense something”. There are five common senses for human beings, namely sighting, hearing, tasting, smelling, and touching. From those senses, signals pass from the body to the brain to response. A device that receives and reacts to environmental information and stimuli is called a sensor [38].

**Figure 1:**
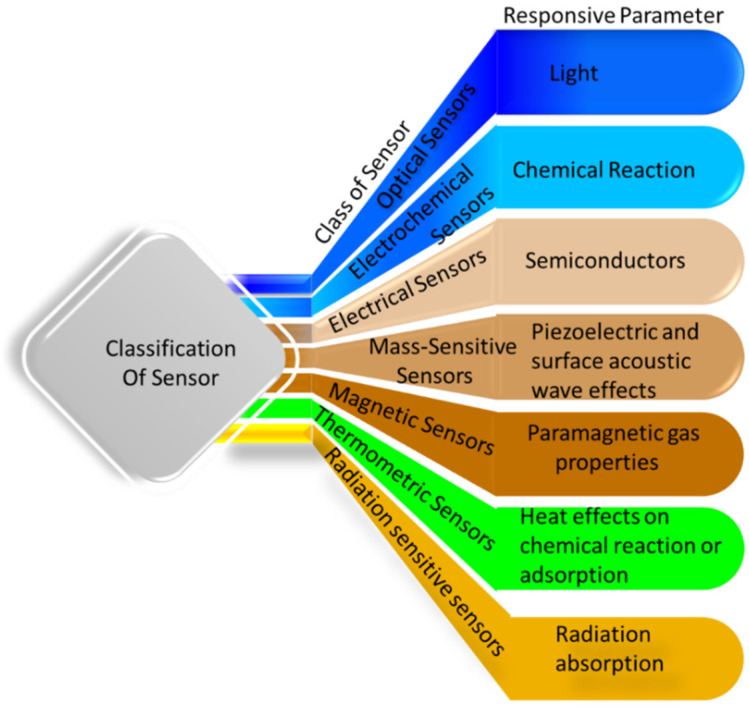
Classification of sensors based on responsive parameters.

**Figure 2:**
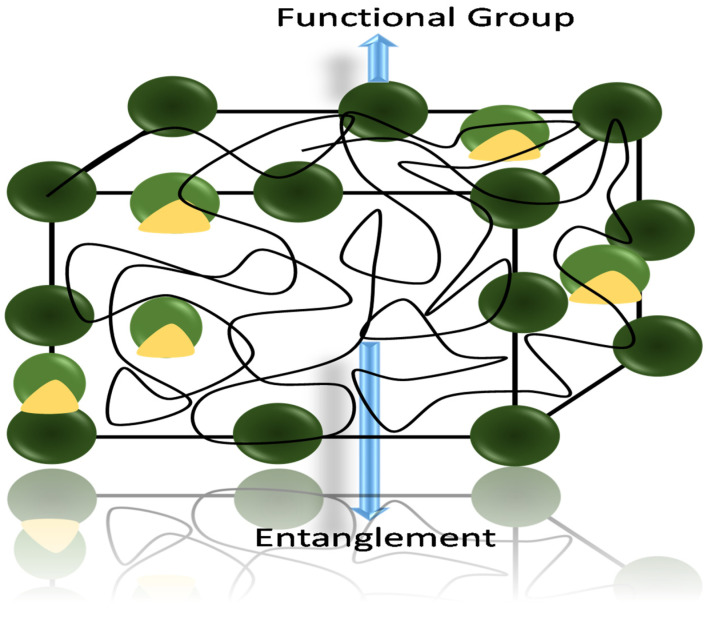
Hydrogel network with cross-linking entanglement and functional groups.

Figure 1 presents the classification of sensors responding to different parameters. A revolution came when conductive single- and multi-network hydrogels were implemented in the field of sensors. The hydrogel possesses functional groups in its structure, which are responsible for the stimuli-responsive nature shown in Figure 2. Due to the presence of different functional groups and the network structure, different stimuli responsiveness appears. When stimulus-responsive hydrogels are exposed to the corresponding stimulus, molecular interactions cause volume-phase transitions that cause sudden network alterations, including swelling, collapse, or solution-to-gel transitions. Thus, the stimulus itself is therefore crucial to the hydrogel response mechanism. For example, the presence of acidic and basic groups in the network shows the responsivity toward the pH of the medium. The pH of the medium changes the mesh size of the network during swelling. Lowering the pH causes protonation of the functional groups (-COOH, -NH_2_), resulting in a decrease in swelling [39].

**Figure 3:**
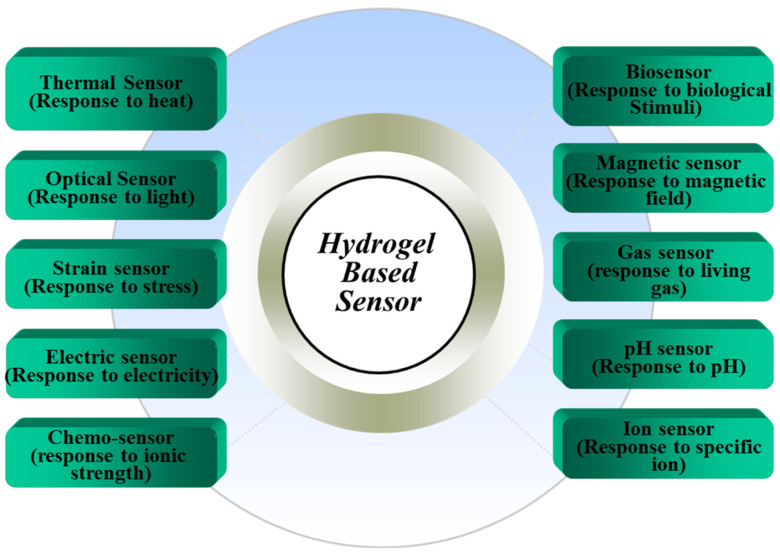
Hydrogel-based sensors [14].

Therefore, based on responsiveness, hydrogel-based sensors can be classified into several types, as shown in Figure 3. Being responsive to a specific parameter, as an output signal a hydrogel exerts pressure by swelling or shows conductivity. The rate of response depends on the hydrogel’s size, shape, composition, cross-linking density, porosity, ionic groups, etc. [38,40]. In this review, a few types of hydrogel-based sensors and their functional mechanisms are introduced. Hydrogels can be classified based on their temperature response into positive and negative thermosensitive types. Positive thermosensitive hydrogels shrink below their upper critical solution temperature (UCST), while negative ones swell below their lower critical solution temperature (LCST). The transition at LCST is influenced by hydrophobic interactions and hydrogen bonding, where increased temperature leads to a loss of water molecules and a collapse of the network. Numerous scientific and technological domains, including food processing, biotechnology, subterranean geochemical research, and marine research, depend on temperature sensors. Therefore, sensors that can accurately measure temperature in a range of environmental circumstances are required. Thus, the abrupt and adjustable entropy-driven collapse of LCST polymeric systems at a specific temperature is garnering a lot of interest despite the abundance of temperature sensors. Poly (acrylic acid), polyacrylamide and poly (acrylamide-co-butyl methacrylate) hydrogels have a characteristic UCST, and N-methylacrylamide, N,N-dimethylacrylamide, and N-isopropylacrylamide) are characterized by LCST [38,41]. Biomolecules such as proteins and glucose can be detected by certain hydrogels. Boronic acid ligands, for instance, are used in glucose-sensitive hydrogels; when glucose binds to these ligands, protons are released, which may be detected [42,43].

**Figure 4:**
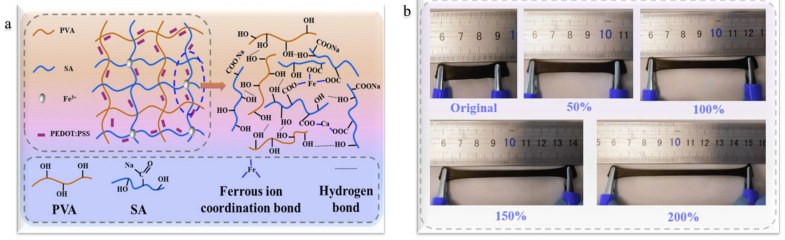
Hydrogel-based soft sensor: (**a**) structure of PVA/SA/PEDOT: PSS (PSPP) hydrogel; (**b**) strain up to 200% [51].

**Figure 5:**
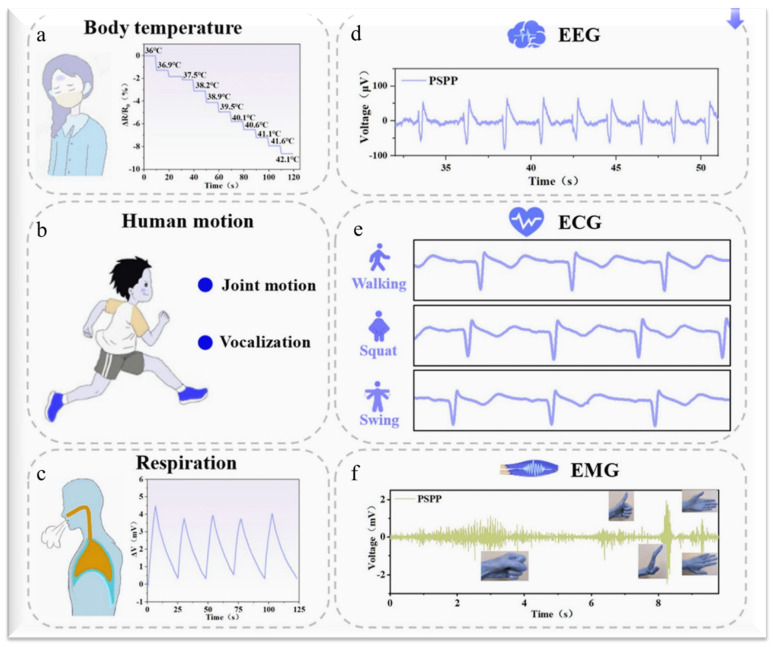
Application of PVA/SA/PEDOT: PSS (PSPP) hydrogel: (**a**–**c**) temperature sensing and (**d**–**f**) physiological signals (EEG, ECG, EMG) sensing [51].

The development of a 3D hydrogel surface-enhanced Raman spectroscopy (SERS) chip for real-time monitoring of pH and glucose levels in sweat is another significant advancement in the hydrogel-based sensing system, reducing the drawbacks of current invasive diagnostic techniques [44]. A hydrogel surface-enhanced Raman scattering (SERS) chip was created by Mingming Chen et al. that can detect very low concentrations of the dangerous food contamination T-2 toxin and provide quick results in as little as five minutes [45]. Silver nanoparticles (AgNPs) are assembled in situ in PVA solution by Ca^2+^, and then they undergo physical cross-linking to create the SERS chip [45]. Through enzymatic processes, enzyme-functionalized hydrogels may also detect glucose and transform it into gluconic acid. Using ion-sensing molecules like calmodulin, which changes the swelling degree according to ion concentration, ion-responsive hydrogels can detect certain ions, such as calcium [46]. Hydrogels are biocompatible and helpful in biomedical applications because of their high-water content and similarity to the natural environment of cells. They can let tiny molecules flow through and shield sensor components from undesirable interactions, but the structure of the gel may make it difficult for bigger molecules to disperse. Additionally, hydrogels can stabilize biosensing components, guaranteeing their continued activity and ability to interact selectively with target molecules, so enabling their application in sensors. When interacting with target substances, they may be tailored for a variety of applications, such as sensing proteins and other biomolecules, and they can develop new sensing systems based on volume changes [47,48,49]. Materials like metal nanowires or carbon nanotubes (CNTs) can be included in the hydrogel matrix to improve the electrical conductivity of hydrogels. This raises the sensor’s total strain sensitivity in addition to improving its electrical characteristics. Together, these processes allow hydrogels to perform well as strain and pressure sensors, which makes them appropriate for a range of uses, such as wearable technology and human–machine interfaces [50]. Recently, a few multifunction sensors have been developed that can simultaneously act as temperature, strain, and bio sensors. Junjie Zheng et al. reported low-cost polyvinyl alcohol (PVA) and sodium alginate (SA) hydrogel-based sensors for strain, temperature, and electrophysiology sensing [51]. They used poly(3,4-ethylenedioxythiophene)/polystyrene sulfonate (PEDOT: PSS) as the conductive material to achieve multifunctional detection. Figure 4a presents the PVA/SA/PEDOT: PSS (PSPP) hydrogel which was created using a freeze–thaw method, making it flexible and able to sense temperature, strain, and electrical signals. The addition of Fe^3+^ ions helped strengthen the hydrogel’s structure and enhance its electrical properties, allowing it to detect changes effectively [52]. The material’s exceptional conductivity (256 mS/m) and flexibility enable the sensor to adjust to the different forms and motions of the human body. The hydrogel’s structure was enhanced by the freeze–thaw procedure, which made it more stable and crystalline, both of which are critical for its functionality. When force is applied, the hydrogel’s porous structure helps absorb energy, increasing its toughness and creating channels for ion transport. The hydrogel’s exceptional mechanical qualities, such as its elongation of 334% and strength of 118.8 kPa, enhance its performance and longevity in a range of sensing applications. The elongation up to 200% and change in stress are shown in Figure 4b. The hydrogel can identify minute strains as low as 4%, demonstrating its remarkable sensitivity to strain. Its mechanical characteristics, which enable it to react rapidly to changes in tension or form, are responsible for its sensitivity. The sensor may be used to monitor dynamic activities because of its quick reaction time of 2.2 s under 10% strain. The hydrogel’s capacity to alter resistance in response to temperature changes is what gives it its temperature-sensing properties, as shown in Figure 5a–c. Its ability to detect temperature changes is demonstrated by decreasing resistance as the temperature rises. With a high sensitivity of −27.43 Ω/K and 0.729 mV/K, the sensor shows promise for use in monitoring respiration and body temperature. As seen in Figure 5d–f, hydrogel is also useful for tracking physiological signals such as ECG, EMG, and EEG. Accurate signal detection depends on its ability to adapt tightly to the skin due to its skin-friendly nature, which lowers motion artefacts and contact impedance. It is appropriate for ongoing health monitoring because of this feature [51].

## 3. Hydrogel-Based Soft Display

**Figure 6:**
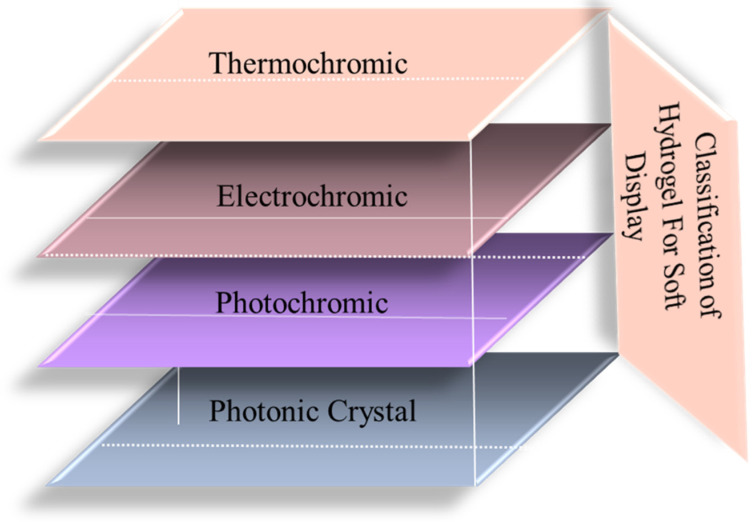
Classification of hydrogel for soft display.

Soft displays are a viable choice for upcoming applications because they combine flexibility, stretchability, brilliant color creation, portability, and durability, and constitute a substantial leap in display technology. Presently, hard displays are being updated with more convenient soft displays—liquid crystals, light-emitting diodes, quantum dots, dielectric elastomers, phosphors, and photonic crystals. Conventional display technologies, such as liquid crystals and LEDs, frequently have drawbacks, such as monochromatic output, manufacturing complexity, and toxicity. Soft displays, on the other hand, are more appropriate for a greater variety of applications, as they can produce a whole spectrum of colors and are composed of non-toxic materials [53]. Hydrogel is the latest inclusion in soft displays, where it acts as a functional component. Hydrogels are a great option for soft display applications because of their special qualities, which include their capacity to change color in response to temperature, durability, design flexibility, and potential for integration with smart technology. These characteristics not only improve displays’ usability, but also create new opportunities for creative designs across a range of sectors. Excellent functional reversibility allows the hydrogel to change color repeatedly without losing its efficacy. Based on their properties, hydrogels for display can be classified into four classes, as shown in Figure 6. For applications involving soft screens, where steady performance over time is necessary, this feature is essential. When compared to the current materials, hydrogels exhibit a longer lifespan in terms of discoloration performance. Because of their dependability, they are a solid contender for real-world uses in soft displays, guaranteeing that the screen will continue to work for long stretches of time. Therefore, soft display technologies may make use of hydrogels to create dynamic visual effects. Their ability to change color may be used for creative and striking designs in fashion, advertising, and art installations [54]. The working mechanism of hydrogels in soft display devices depends on their special composition, light-emitting capabilities, mechanical flexibility, and sturdy manufacturing techniques, which make them appropriate for suitable flexible display technologies. Xiaoyu Luo et al. developed 3D-printed hydrogel-based (prepared from viologen and polyvinyl alcohol) soft displays, specifically flexible electrochromic devices (FECDs), for uses in wearable electronics, camouflage, and smart windows [55]. When an electric potential is supplied, electrochromic elements like viologen, which are present in the hydrogel of FECDs, go through reversible redox processes. The hydrogel can change color as a result of this process, giving the display the ability to represent various visual states. For example, at a low voltage of −1.0/1.0 V, the hydrogel can change from colorless to purple, which is important for applications like visual warnings and emergency signaling. Polyvinyl alcohol (PVA) and viologen are combined to create a hydrogel. This combination improves the hydrogel’s mechanical qualities and printability, enabling smooth integration throughout the 3D-printing process. The resultant material is flexible and long-lasting, and retains strong electrochromic performance. After prolonged usage, the hydrogel-based FECDs show no performance loss and outstanding cycle stability. For instance, they retain strong visual contrast even after 5000 bending cycles and exhibit less than a 5% decrease in electroactivity after 10,000 s of operation. The displays’ long-term dependability is guaranteed by their durability [55]. Yawen Xu et al. prepared double network composites made from polyacrylamide (PAAm) and calcium alginate, cross-linked with vinyl-modified Ce-doped yttrium aluminum garnet (YAG:Ce-VTES) phosphors [56]. This combination enables the optical qualities and mechanical robustness required for display applications. The YAG:Ce phosphors have two functions: they are light-emitting centers and cross-linkers. These phosphors produce white light when stimulated by a blue backlight, which is necessary for the operation of the display. These phosphors’ incorporation into the hydrogel matrix improves the stability and luminous efficiency of the light that is released.

**Figure 7:**
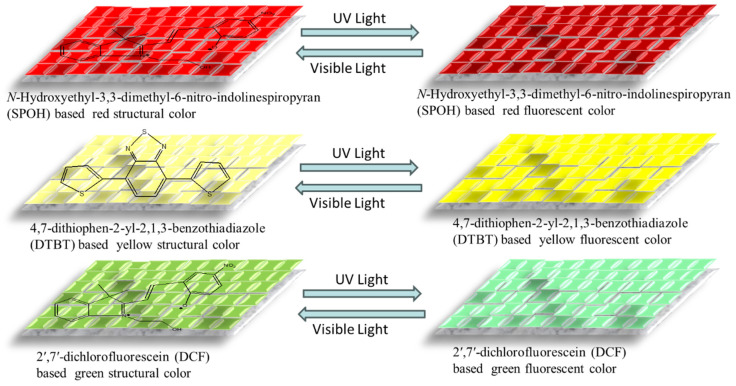
Display mechanism diagram of the structural–fluorescent color photonic crystal hydrogel in the structural color mode and fluorescence color mode.

The hydrogels have a fracture elongation of up to 600%, demonstrating exceptional toughness and stretchability. For flexible displays to bend and stretch without losing their usefulness, this great elasticity is essential. The concentration of YAG:Ce phosphors in the hydrogel can be tuned to modify the mechanical characteristics. By tuning the YAG:Ce-VTES concentration, the hydrogels’ fluorescence intensity is managed. In display applications, this tunability is important for attaining the appropriate brightness and color quality. According to a previous study, the emission wavelength stays constant under mechanical stress, even while the fluorescence intensity drops with increasing strain [56]. Zihan Liu et al. developed a multicolor structural–fluorescent CdS-PEGDA photonic crystal hydrogel (SFC-CPH) with a dual display mode [57]. Prior to being doped into poly (ethylene glycol) diacrylate (PEGDA) hydrogel, CdS colloidal particles were first created using a hydrothermal process. The color change processes upon UV–Visible light exertion on hydrogel in a soft display are depicted in Figure 7. The CdS particles create a photonic crystal structure that produces structural colors. The photonic band gap, which in turn influences the color exhibited, may be controlled by varying the size of these particles. This improves their visibility by producing vibrant structural colors that are less reliant on the viewing angle. Bright fluorescent colors are produced when the fluorescent molecules incorporated in the PEGDA hydrogel are subjected to ultraviolet (UV) light. The chemical structure of fluorescent molecules may be exploited to produce several colors, including green and yellow fluorescence. When exposed to visible light, the hydrogel can make the change between the structural and fluorescent color modes. Users can select the mode that best suits their needs thanks to its dual-mode functionality, which enables a wide range of applications in displays [57].

## 4. Hydrogel-Based Soft Battery and Supercapacitor

**Figure 8:**
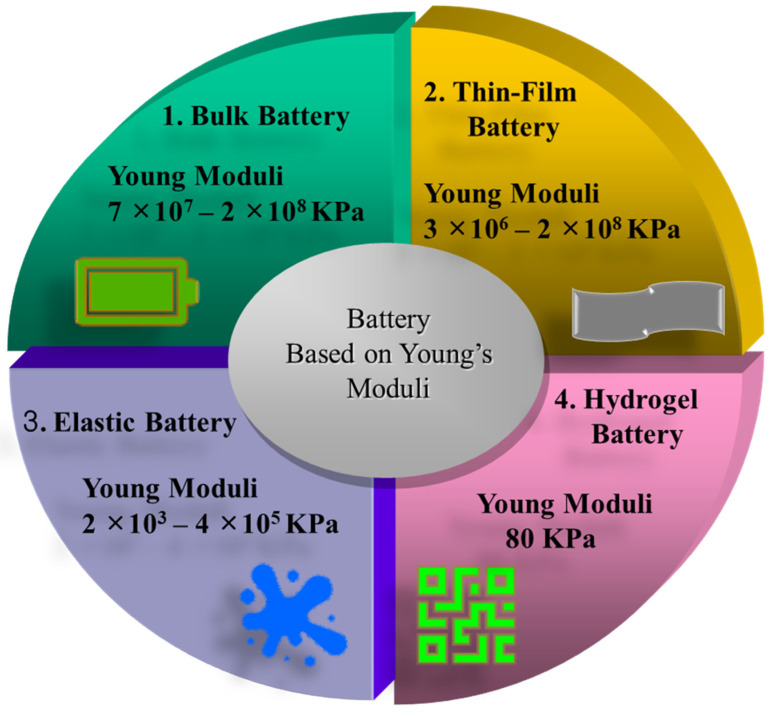
Different types of battery based on Young’s moduli [60].

Batteries and supercapacitors are energy devices made of both bulk and soft materials. Hydrogels help energy storage systems be more flexible and mechanically stable. For applications where electronics must be able to tolerate stretching and bending without losing functionality, this is essential. Energy storage systems can use hydrogels for a variety of purposes. They can be employed as current collectors, binders, separators, electrodes, and electrolytes. Performance may be enhanced by integrating many components into a single device, thanks to its multifunctionality [58]. Many conventional batteries are made from metal, non-metal, carbon nanotubes, conductive polymers, and rigid composite materials, which have high Young’s moduli ranging from 10^2^ to 10^8^ kPa. Because of their rigidity, they are not appropriate for uses like integrating with biological tissues that call for softness and flexibility [59]. The challenges of high rigidity, poor conductivity, morphological limitations, dehydration issues, and biocompatibility limit the usability of batteries in biomedical engineering and motivate the search for new soft materials. Hydrogel is the better candidate for the incorporation of a biocompatible and biodegradable soft battery with lower Young’s moduli. The different classes of batteries have different Young’s modulus values, shown in Figure 8, where the hydrogel-based battery possesses the lowest value, of 80 Kpa, indicating flexibility. Hydrogels based on carbon, for example, have demonstrated encouraging outcomes in terms of energy density and specific capacitance. A specific capacitance of around 1247 Fg^−1^ and energy densities of roughly 43 Wh kg^−1^ were observed in one research study, suggesting that they might be used in flexible supercapacitors [60]. Hydrogel can be employed in batteries as an electrolyte, electrode, membrane, and gelator along with metal components [61]. Hydrogel is even replacing metal in batteries, resulting in the metal-free battery being a revolutionary innovation.

**Figure 9:**
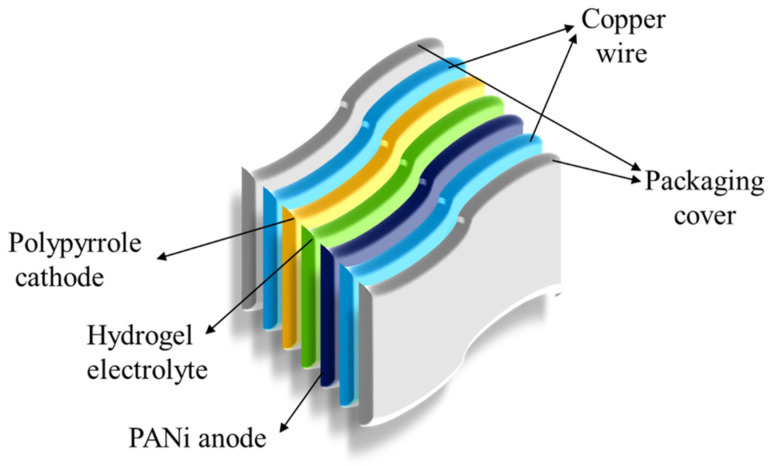
Metal-free hydrogel-based battery.

Abhishek Paudel et al. reported eco-friendly metal-free ammonium ion batteries consisting of a polyaniline anode, polypyrrole cathode, and xanthan gum–ammonium sulphate ((NH_4_)_2_SO_4_) hydrogel electrolytes, as shown in Figure 9 [62]. The battery shows a capacity of 44.321 mA h g^−1^ and retains 74.56% capacity after 100 cycles. Strong mechanical strength and flexibility are demonstrated by the battery’s ability to function well even when bent or twisted, opening the door for biodegradable electronics. Xanthan gum is particularly beneficial due to its biodegradable nature and ability to enhance water retention, which is essential for maintaining ionic conductivity in the electrolyte. The amount of ammonium sulphate in the hydrogel has a major impact on its ionic conductivity. The study discovered that the maximum ionic conductivity, which is essential for the effective movement of ammonium ions (NH_4_^+^) during battery operation, is obtained at a concentration of 3 m of (NH_4_)_2_SO_4_. When ionic conductivity is high, electrochemical performance is enhanced [62].

**Figure 10:**
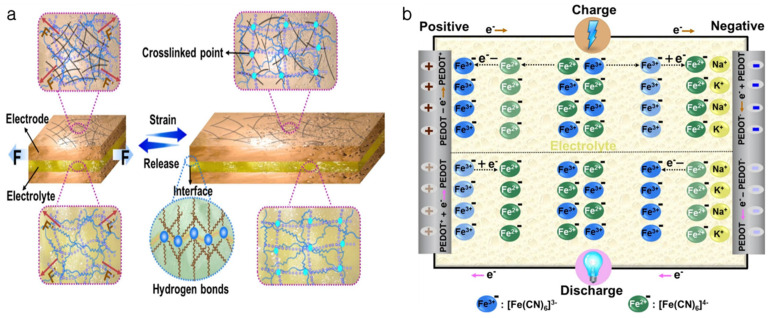
Hydrogel-based supercapacitor: (**a**) the stable network structure of hydrogel electrode and electrolyte during the stress–strain process, F = force; (**b**) the charge–discharge mechanism of a hydrogel capacitor. (Reused with permission from reference [65]).

Innovative energy storage technologies called soft supercapacitors—in particular, wearable supercapacitors, or WSCs—were created to satisfy the rising need for portable and adaptable electronic applications. The electrode–electrolyte interaction and electrode materials utilized have a major impact on supercapacitor performance. Enhancing the electrochemical qualities needed for wearable applications requires the use of soft electrode materials, such as composites made of metal, polymers, conductive polymers, hydrogels, and carbon. Due to their self-adhesive qualities, hydrogel materials are becoming more and more popular. This makes them appropriate for conformal interaction with biological tissues. This is especially important for applications involving health monitoring because the soft device must stay in touch with the skin [63]. The hierarchical porosity structure of conductive hydrogels (CHs) greatly improves ionic and electrical conductivity. This is necessary for supercapacitors to store energy and transfer charges efficiently. The hydrogel’s linked network of conductive elements makes it easier for ions and electrons to travel, which is essential for the charging and discharging processes. When a voltage is applied, ions diffuse through the hydrogel’s hierarchical pores, while electrons travel through the conductive network. The supercapacitor’s total efficiency is increased by this dual transfer method. Moreover, hydrogels can gain high specific capacitance values because of their large surface area and the ability to accommodate a significant amount of electrolyte. For instance, specific capacitance values as high as 885 mFcm^−2^ have been demonstrated by supercapacitors using hydrogel electrodes, demonstrating their efficacy in energy storage applications [64]. Juan Zeng et al. prepared soft supercapacitors made from double-network hydrogel electrodes and electrolytes that can stretch and compress without extra materials, ensuring good energy output [65]. Figure 10a shows the structure of both the hydrogel electrode and electrolyte, which feature a three-dimensional (3D) cross-linked network. The electrode consists of a dual-network hydrogel matrix made of sodium alginate (SA) and polyacrylamide (PAM). The electrode can tolerate a range of deformations without losing its performance, thanks to the strong framework this combination offers, which also improves mechanical stability and flexibility. A linked conductive network is produced when carbon nanotubes (CNTs) are incorporated into the hydrogel matrix. Stable electronic channels are maintained by this network, improving the electrode’s overall conductivity. The CNTs cooperate to mitigate the effects of mechanical stress, ensuring that the electrode retains its performance under deformation. PEDOT: PSS, an active material renowned for its superior electrochemical qualities, is integrated into the electrode. This conductive polymer improves the electrode’s ionic conductivity and charge storage capacity, enabling effective energy transfer while the device is operating. Like the electrode, the electrolyte is made of a dual-network hydrogel matrix, mostly consisting of sodium alginate (SA) and polyacrylamide (PAM). This composition improves the supercapacitor’s overall performance by offering an adaptable and stable environment for ionic transport. The electrolyte possesses 0.5 M Na_2_SO_4_ as an electrolyte salt, which significantly enhances ionic conductivity. Furthermore, the hydrogel electrolyte’s charge storage capacity is increased by the presence of the redox couple K_3_[Fe(CN)_6_]/K_4_[Fe(CN)_6_], which improves its efficiency in energy transmission during operation. Figure 10b exhibits the charge–discharge mechanism governed by the hydrogel in the supercapacitor. Electrical double-layer capacitance and Faradaic processes are both involved in the charge storage mechanism. Charge buildup results from Faradaic reactions that take place at the conductive polymer’s (PEDOT) surface, where cations from the electrolyte are absorbed and electrons are transported. By retaining charge at the interface between the electrode and electrolyte, the electrical double-layer capacitance adds to the total capacitance. Fast Faradaic reactions are a hallmark of the pseudocapacitive phenomenon used by the supercapacitor. The hydrogel’s redox pair, which specifically involves the oxidation and reduction of ions like PEDOT and [Fe(CN)_6_]^4−^, facilitates this action. Effective charge storage and release are made possible by the oxidation of [Fe(CN)_6_]^4−^ during charging and the reduction that happens during discharging [65].

## 5. Hydrogel-Based Soft Circuits

**Figure 11:**
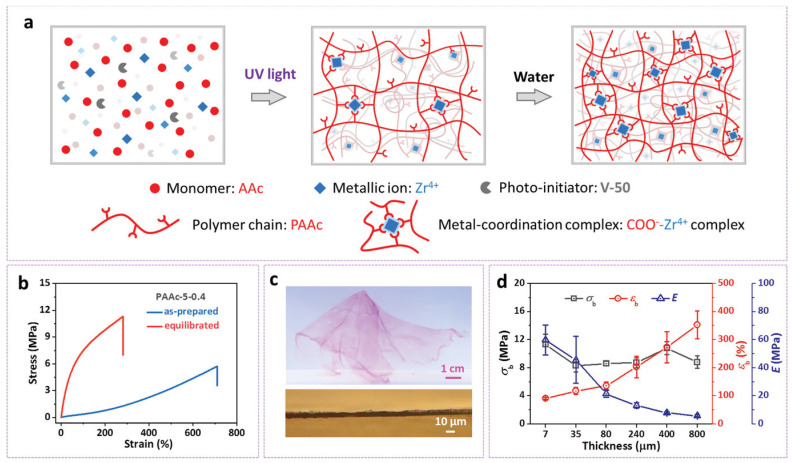
(**a**) Synthesis of PAAc-Zr hydrogels, (**b**) tensile stress curve of the hydrogels, (**c**) cross-sectional close view of hydrogel, and (**d**) mechanical properties of hydrogel with a 7 μm thickness. (Reused with permission from reference [77]).

Hydrogel–based soft circuits represent a significant advancement in flexible and bio-compatible electronics, and are particularly suited for applications in wearable devices, soft robotics, and bioelectronics. These circuits leverage the unique properties of hydrogels, such as ionic conductivity and mechanical compliance, which enable them to conform to dynamic environments while maintaining functionality [66,67,68]. However, challenges persist, including low electrical conductivity (10^−3^–10^1^ Sm^−1^), release of ions to body fluids, and mechanical instability, which can hinder device performance [67,69]. To address these limitations, researchers explore strategies such as incorporating conductive fillers, applying cross-linking techniques, and developing composite materials that enhance the circuits’ electrical and mechanical properties [70]. The potential applications of hydrogel-based soft circuits span wearable sensors for physiological monitoring, soft robotics for adaptable movement, and bioelectronics for interfacing with biological systems, underscoring their promise in advancing electronic technologies [71]. These circuits fall into three main categories: stretchable circuits that use conductive hydrogels or elastomers to function effectively under deformation; flexible circuits, defined by their thin, pliable substrates ideal for conformable sensors and displays; and bio-integrated circuits, which integrate smoothly with biological tissues by utilizing hydrogels for their biocompatibility and ionic conductivity [72]. Hydrogels naturally have distinctive features, such as high ionic conductivity and mechanical compliance, enabling them to adapt to changing environments [36]. Despite their promising potential, several challenges persist, such as the relatively low electrical conductivity of pure hydrogels and mechanical degradation over time, especially under mechanical stress. Recent advancements have aimed to address these limitations, such as integrating conductive fillers like graphene, carbon nanotubes, and silver nanoparticles to enhance conductivity, alongside advanced cross-linking methods that improve the hydrogel’s mechanical strength [73]. Composite materials that combine hydrogels with other flexible polymers or metals have also been developed to overcome these weaknesses, creating hybrid systems that balance both electrical performance and mechanical stability. One of the key aspects that makes hydrogels ideal for soft circuits is their ionic conductivity, where ions within the hydrogel move in response to an applied electric field, making them effective for bioelectronics and wearable sensors. In these devices, hydrogels conduct electricity through ion transport, rather than electron flow, which is characteristic of conventional electronic materials. This allows hydrogels to function in moist or liquid environments, which is essential for applications in medical devices that interact directly with biological tissues. By adding conductive elements like PEDOT: PSS, conventional hydrogels may be altered for dual conductivity (ionic/electronic). A novel approach that has the potential to transform bioelectronics is the active processing and amplification of bioelectronic signals using semiconductor hydrogels [74]. Professor Li et al. developed a new n-type semiconducting single network hydrogel that opens new bioelectronics possibilities by fabricating logic circuits [75]. These semiconducting hydrogels exhibit remarkable biocompatibility, low operating voltage, and great sensitivity when utilized in OECTs. Multiple-network hydrogels, which offer enhanced qualities including stretchability and bioadhesiveness, were created by fusing semiconducting hydrogels with other networks [75]. Addressing these issues should be the main goal of future research, particularly the creation of high-performing p-type semiconducting hydrogels for improved signal processing effectiveness in bioelectronic devices. Han et al. fabricated a hydrogel-based ionic circuit on a polydimethylsiloxane (PDMS) microchip with high tensile strength and long-term stability [76]. Diallyldimethylammonium chloride and sodium styrenesulfonate were used as monomers for n-type and p-type charged gels. The author claimed that this could be used in different bio-applications. Zheng’s group synthesized hydrogel films by polymerizing precursor solutions of acrylic acid in the presence of Zr^4+^ ions that form robust carboxyl-Zr^4+^ coordination bonds [77]. Thus, they showed high mechanical performance with a tensile breaking stress of 0.4–11.9 MPa, a breaking strain of 45–390%, Young’s modulus of 0.07–186 MPa, and a tearing fracture energy of 0.07–8.9 kJm^−2^ with a water content of 45–95%, as shown in Figure 11. For potential applications, the author utilized a hydrogel with a kirigami structure mixing liquid metal, and found high sensitivity due to its thin and flexible properties.

The ionic conductivity of hydrogels is governed by the movement of ions through a polymer network, where the polymer matrix provides mechanical support while allowing ions to migrate in the presence of an electrical potential. This behavior is fundamentally different from traditional metallic conductors that rely on electron flow. The ionic conductivity of a hydrogel can be described by Equation (1) [78].

σ = nqμ(1)

where σ, n, q, and μ represent ionic conductivity, ion concentration (number of ions per unit volume), charge of the ion, and ion mobility (the velocity of ions under an applied electric field), respectively. The water content influences the ionic conductivity in the hydrogel, as water serves as the medium for ion transport. The ability of hydrogels to retain water is crucial, as hydration increases ion mobility, thus enhancing conductivity. However, challenges like swelling and dehydration can affect performance, especially under varying environmental conditions [79]. Bowen Yao et al. prepared a conducting polymer hydrogel by PEDOT: PSS, which demonstrated conductivity up to 1200 Scm^−1^ [80]. This property was attributed to the H_2_SO_4_ chemical treatment during the synthesis process.

## 6. Hydrogel-Based Soft Robots

**Figure 12:**
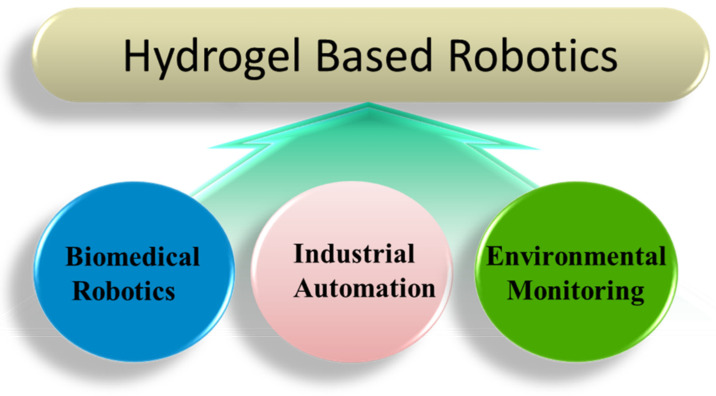
Classification of hydrogel-based robotics.

Soft robotics is the field of robotics where soft, flexible materials like hydrogels and elastomers are used to create robots that are comparatively safer and more capable of functioning in complex tasks in a variety of environments. Due to their tunable smart properties, softness, elasticity, and modification with nanomaterials, hydrogels have been one of the efficient candidates for the manufacturing of soft robots [81]. Based on applications, hydrogel-based soft robots can be classified into three classes, as shown in Figure 12.

**Figure 13:**
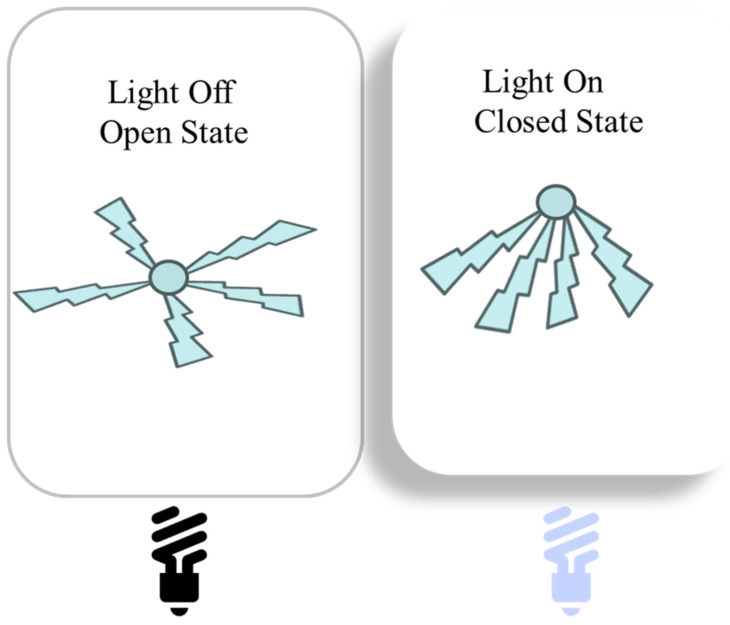
Effect of switching a light on and off on the movement of a JSMR under visible light.

Hydrogel contributes to two main components of soft robots, namely actuators and sensors, which facilitate robots to provide safer and more adaptable services. Hydrogels are modeled after biological systems, which combine soft and stiff components. Soft robots are more flexible and safer because of this biomimetic technique, which also makes them better suited to a range of operating settings and human interactions. Hydrogels’ softness lowers the possibility of harm during intimate human contact. Hydrogels in soft robotics work through a variety of methods, including biomimetic design, improved sensing capabilities, environmental adaptation, responsive actuation, and sophisticated manufacturing techniques. Together, these characteristics enhance the usefulness and adaptability of hydrogel-based continuum soft robotics, opening the door for creative uses in a variety of industries. In reaction to external stimuli, including temperature, pH, light, and electric fields, hydrogels can alter their volume or form. Because of this characteristic, soft robots may act as actuators and carry out intricate movements and duties. For example, hydrogels provide fine control over the robot’s behaviors by expanding or contracting in response to various solutes or solvents. Hydrogels are used in soft robots to improve their senses. Numerous sensors that measure pressure, humidity, strain, and other environmental variables may be included in hydrogels. Robots with this multimodal sensing capacity can collect data in real time and react appropriately, which makes them appropriate for use in environmental monitoring and healthcare applications [82]. Moses Gladson Selvamuthu et al. developed a double-network gel-based soft inchworm robot, which is cost-effective, and easy to use and dispose of [83]. The voltage on the legs of the robot can be changed to regulate its movement, enabling a variety of speeds and motion techniques. Water seepage in DN gel affects the robot’s functioning, and for best results, it must be regularly rehydrated [83]. Chao Yin et al. fabricated a jellyfish-like miniature soft robot (JMSR) made of poly(N-isopropylacrylamide) (PNIPAM), PEDOT: PSS, and carbon nanotubes (CNTs) [84]. This combination allows the hydrogel to be actuated by visible light, temperature, and the concentration of CNT changes, which is essential for its actuation. The robot shows movement, i.e., swimming, walking, and jumping, followed by the transportation of tiny objects. The hydrogel behaves differently depending on the temperature. The hydrogel absorbs water and expands, showing itself hydrophilic below its lower critical solution temperature (LCST) of 32 °C. The change in state of hydrogel in the robot upon applying visible light is exhibited in Figure 13. When the hydrogel is exposed to visible light, the CNTs absorb light energy, causing a rapid increase in temperature. The hydrogel bends significantly because of this temperature increase, enabling the robot to swim and move in different ways. The hydrogel’s ability to efficiently transform light energy into mechanical motion is demonstrated by the bending speed, which can reach up to 65.72°/s. The light source may be turned on and off to regulate the bending motion of the NIPAM/CNT hydrogels. When the light is on, the temperature increases, and then the hydrogel undergoes bending. The hydrogel is restored when the switch of light is off. This mechanism facilitates the JMSR for various movement modes, including swimming, walking, and jumping. Periodically switching the light on and off moves the JMSR upward, carrying a small object with it. Thus, the hydrogel is a flexible actuator for the soft robot, as it can be made to change its deformation using light [84].

## 7. Scope of Improvement and Future Prospects

Hydrogel materials have drawbacks despite their benefits, including poor tensile strength and constrained working windows. For hydrogel-based energy storage systems to be more commercially viable, these issues must be resolved [58]. Hydrogel-based electronics may face structural damage at lower temperatures (especially below zero) because water within the hydrogel freezes, leading to a loss of mechanical integrity. Improvement in mechanical integrity may bring the solution. Water on the hydrogel or skin surface may make it harder for the device to stick to the skin. For health monitoring applications, this might result in problems with data reliability and even device separation while in operation. When subjected to high temperatures, hydrogel-based soft electronics frequently show a decline in mechanical resilience. Their stretchability and flexibility are affected, which limits their application. Temperature variations can have a substantial impact on the conductivity of sensing components in hydrogels, resulting in unstable sensing performance. The accuracy of health monitoring data may be jeopardized by this unpredictability. To mitigate this problem, stimuli responsivity can be improved so that the hydrogel can be adjusted with temperature changes. In a word, there is a lot of scope for advancement in hydrogel-based wearables and soft electronics, including in the areas of material science, durability, structural design, and user experience.

## 8. Conclusions

In this study, the inclusion of hydrogels in different soft electronics like sensors, displays, robots, batteries, supercapacitors, and circuits has been discussed. The functional mechanism of hydrogels in soft electronics is illustrated to highlight their importance. Most of the applications of hydrogel are in sensors, where the properties of hydrogel are used to make a specific sensor device. Due to their flexibility and stretchability, hydrogel-based soft displays are becoming popular. Hydrogel-based soft circuits present an exciting frontier in the field of flexible electronics, offering distinct advantages like biocompatibility, mechanical compliance, and ionic conductivity. While challenges related to electrical conductivity and mechanical degradation persist, recent advancements in composite materials, conductive fillers, and cross-linking methods are addressing these issues. As research progresses, hydrogel-based soft circuits are expected to play a pivotal role in the future of wearable electronics, soft robotics, and bioelectronic interfaces, with the potential to revolutionize fields like medicine and sustainability. Hydrogel technology recently replaced the metal-ion battery, which is the most revolutionary advance. Due to the improvement in conductivity, hydrogels are used in soft circuits. Different levels of actuation biocompatibility of hydrogels have opened the door for soft robots mimicking biological activities. Despite this progress, hydrogels need some major improvements, including improved mechanical strength to adjust to different situations and conductivity.

## Figures and Tables

**Table 1:** Comparison between traditional and hydrogel-based soft electronics.

Types of Electronics	Types of Materials	Components	Performance/Feature	References
Sensor	Liquid-metal-based pressure sensor	Metals, metal oxides, silicon wafers, glass, ceramics.	Low sensitivity, rigid; stretchability < 1%.	[21]
	Soft hydrogel-based pressure sensor	Single or multi-layer hydrophilic polymer networks (polyvinyl alcohol, chitosan etc.)	High sensitivity, ultra-stretchable (up to 300–1000% strain.	[22]
Display	Traditional display	Amorphous silicon (a-Si), low-temperature polysilicon (LTPS), or metal oxides	Resistant to environmental stress	[23]
	Hydrogel-based display	Water-rich polymer networks that provide flexibility, stretchability and self-healing, electronic and optoelectronic functionalities	Can expand over 1500% without losing function, self-healable.	[24,25]
Battery	Traditional battery	Metal ions like lithium-ion, lead-acid, nickel-cadmium etc.	Exhibit better cycle stability, have higher energy density.	[26]
	Hydrogel-based battery	Polymers, polyaniline anode and polypyrrole cathode, ammonium cations (NH_4_^+^), metal ions.	Exhibit better cycle stability and resistance to degradation.	[27]
Supercapacitor	Traditional supercapacitors	Activated carbon, graphene, or carbon nanotubes,	Longer cycle life	[28,29]
	Hydrogel-based supercapacitors	Polyvinyl alcohol, polypyrrole, carbon nanotube films	Longer cycle life with enhanced stability	[30]
Circuit	Traditional circuits	Silicon-based semiconductors, metallic conductors, organic transistors.	Higher electronic conductivity, more stable	[31,32]
	Hydrogel	Conductive hydrogels, polymeric networks with ionic or electronic conductors, metal-polymer hydrogel.	Rely on ionic conductivity, require hydration to maintain performance	[33]
Robot	Traditional robots	Steel or aluminum frames, risk of injury on collision	Deformation is resisted by stiff connections.	[34,35]
	Hydrogel-based robots	Hydrophilic polymer matrices	Can undergo large strains (100–1000%)	[36,37]

## Data Availability

No new data was created or analyzed in this study.
